# Sex-dependent effects of canagliflozin and dapagliflozin on hemostasis in normoglycemic and hyperglycemic mice

**DOI:** 10.1038/s41598-023-28225-8

**Published:** 2023-01-17

**Authors:** Natalia Marcińczyk, Tomasz Misztal, Ewa Chabielska, Anna Gromotowicz-Popławska

**Affiliations:** 1 Department of Biopharmacy and Radiopharmacy, Medical University of Bialystok, 15-222 Bialystok, Poland; 2 Department of Physical Chemistry, Medical University of Bialystok, 15-089 Bialystok, Poland

**Keywords:** Pharmacodynamics, Experimental models of disease, Preclinical research, Cardiovascular diseases, Platelets, Platelets, Thrombosis

## Abstract

Sodium-glucose cotransporter 2 inhibitors (SGLT2i) are antihyperglycemic drugs that decrease mortality from cardiovascular diseases. However, their effects on hemostasis in the cardioprotective effects have not been evaluated. Therefore, the effects of canagliflozin (CANA, 100 mg/kg, *p.o.*) and dapagliflozin (DAPA, 10 mg/kg, *p.o.*) on the parameters of hemostasis were investigated in female and male normoglycemic and streptozotocin (180 mg/kg, *i.p.*)-induced diabetic mice. CANA and DAPA reduced platelet activity in thrombus in male and female mice both normoglycemic and diabetic. CANA decreased thrombus formation in diabetic male mice, and platelet activation to ADP in diabetic female and male mice. Activation of fibrinolysis was observed in female mice, both normoglycemic and diabetic. DAPA reduced thrombus formation in diabetic male and female mice, and decreased platelet activation to ADP and fibrin formation in diabetic male mice. DAPA increased fibrin formation in normoglycemic female mice and activated fibrinolysis in diabetic female mice. CANA and DAPA exerted sex-specific effects, which were more pronounced in hyperglycemia. The antithrombotic effect of CANA and DAPA was more noticeable in male mice and could be due to platelet inhibition. The effect on coagulation and fibrinolysis was not clear since an increased coagulation and fibrinolysis were observed only in female mice.

## Introduction

Sodium-glucose cotransporter 2 inhibitors (SGLT2i) are antihyperglycemic drugs used primarily in the treatment of type 2 diabetes. Examples of these drugs are canagliflozin (CANA), dapagliflozin (DAPA), and empagliflozin. The mechanism of their hypoglycemic action is based on the inhibition of the SGLT2 transporter in the renal proximal tubule, which is responsible for the majority of the reabsorption of filtered glucose. Hence, inhibition of SGLT2 leads to an increase in urinary glucose excretion^1^. Large clinical trials such as Canagliflozin Cardiovascular Assessment Study (CANVAS)^2^ and Dapagliflozin Effect on Cardiovascular Events (DECLARE-TMI 58)^3^ have shown that SGLT2i decrease the mortality from cardiovascular diseases. Therefore, some SGLT2i have been approved in the treatment of heart failure without coexisting diabetes^4,5^. Cardiovascular diseases are often accompanied by disturbances in hemostasis, which may lead to serious complications, in which the thrombotic process plays a key role^6^. The mechanism of the cardioprotective action of SGLT2i has not yet been fully elucidated, but it probably includes anti-inflammatory, antioxidant, and hypotensive effects and an increase in the production of nitric oxide (NO) by the endothelium^7^. The antiplatelet effect of DAPA in patients with type 2 diabetes and stable coronary artery disease (The Effect of Dapagliflozin on Platelet Function Testing Profiles in Diabetic Patients: The EDGE Study)^8^, *Ldlr*^*−*^*/*^*−*^ mice with hypercholesterolemia and healthy volunteers^9^ as well as the in vitro antiplatelet effect of CANA^10^ may suggest the role of hemostasis in the beneficial effects of these drugs on the cardiovascular system. However, clinical trials indicate that the use of DAPA (DECLARE-TMI 58)^3^ or CANA (CANVAS)^2^ did not reduce the overall risk of myocardial infarction (MI) and ischemic stroke. Due to these inconclusive results, the effects of SGLT2i on the hemostatic system cannot be clearly defined. To date, no studies have been conducted to determine the direct effect of SGLT2i on the thrombotic process and hemostasis. Therefore, this study aimed to investigate the effect of two SGLT2i that differ significantly in pharmacokinetics and pharmacodynamics: CANA and DAPA, on the experimental thrombotic process and selected hemostasis parameters. DAPA is characterized by higher selectivity toward SGLT2 than CANA, which also has a low affinity to SGLT1^11^. Furthermore, there are differences in biological activity between them, such as the effect on vasodilation in isolated mouse heart^12^, secretion of interleukin-6 in HUVECs^13^, and effects on liver function parameters in diabetic patients^14^. The effects of CANA and DAPA on hemostasis were evaluated in normoglycemia and hyperglycemia. Due to the significant effect of sex hormones on the pathogenesis of cardiovascular diseases and hemostasis^15^, the more pronounced benefits of SGLT2i in the treatment of heart failure in women^16^, and potential sex-disparate effects of SGLT2i on major adverse cardiac events^17^, studies were conducted on female and male mice.

## Materials and methods

### Animals

Male and female C57BL6 mice (weighing 18–23 g) were used in this study. They were randomly divided into 12 groups:

- Contr Norm-female—female normoglycemic mice treated with 5% water solution of gum arabic;
- Contr STZ (streptozotocin)-female—female diabetic mice treated with 5% water solution of gum arabic;
- CANA Norm-female—female normoglycemic mice treated with CANA;
- CANA STZ-female—female diabetic mice treated with CANA;
- DAPA Norm-female—female normoglycemic mice treated with DAPA;
- DAPA STZ-female—female diabetic mice treated with DAPA;
- Contr Norm-male—male normoglycemic mice treated with 5% water solution of gum arabic;
- Contr STZ-male—male diabetic mice treated with 5% water solution of gum arabic;
- CANA Norm-male—male normoglycemic mice treated with CANA;
- CANA STZ-male—male diabetic mice treated with CANA;
- DAPA Norm-male—male normoglycemic mice treated with DAPA; and
- DAPA STZ-male—male diabetic mice treated with DAPA.

Before laser-induced thrombosis and blood collection, all mice were anesthetized with a single injection of ketamine and xylazine mixture (120 mg/kg, *i.p.*, Ketamina 10%, Biowet, Poland; 12.5 mg/kg, *i.p.*, Xylapan, Biowet, Poland). After the experiments, the mice were euthanized by cervical dislocation.

All methods are in accordance with ARRIVE guidelines.

### Diabetes induction

At the 1st day of the experiment STZ or citrate buffer were injected to mice. Diabetes was induced in the STZ groups using a single intraperitoneal injection of STZ at a dose of 180 mg/kg. The Norm groups were injected with an equal volume of a citrate buffer. The blood glucose level was measured 14 days and 28 days after the first STZ injection (at the 14th and 28th day of the experiment respectively). Diabetes was defined as a blood glucose level of > 200 mg/dl, which was measured using an Optium Xido glucometer and Optium Xido glucose test strips (Abbott, United States). The beta-hydroxybutyrate (βHB) level, a marker of the production of ketone bodies, was measured after 14 days of DAPA, CANA, or gum arabic administration (at the 28th day of the experiment) using an Optium Xido glucometer and Optium Xido test strips for measuring β-ketone bodies (Abbott, Unites States).

### CANA and DAPA administration

The mice were administered CANA (Invocana, Janssen Pharmaceuticals, Belgium) or DAPA (Forxiga, Astra Zeneca, UK) using an oral gastric tube, once daily at doses of 100 and 10 mg/kg, respectively, in a volume of 3 ml/kg in 5% water solution of gum arabic for 14 days (from 14 to 28th day of the experiment). The STZ groups were administered CANA and DAPA 14 days after the STZ injection. Doses were selected based on the pharmacokinetics of CANA and DAPA in mice^18^. The Contr Norm and Contr STZ groups received an equal volume of a 5% water solution of gum arabic (from 14 to 28th day of the experiment).

### Confocal microscopy observation

In the experiments of laser-induced thrombosis, and visualization of fibrin net, a fixed-stage microscope Zeiss Axio Examiner Z1 (Carl Zeiss Microscopy GmbH, Germany), a confocal scanner unit (CSU-X1, Yokogawa Electric Corporation, Japan), and a W Plan-Apochromat 20 × /1.0 water immersion objective (Carl Zeiss Microscopy GmbH) were used. SlideBook 6.0 (Intelligent Imaging Innovations, Inc., United States)^19^ was used to analyze the recordings and images.

### Laser-induced thrombosis in the mesenteric vein and the assessment of platelet activity based on the platelet endothelial cell adhesion molecule (PECAM-1)/thrombus ratio

To evaluate the effects of CANA and DAPA on the experimental model of thrombosis, laser-induced thrombosis was performed as described previously^20^. In brief, 5 min before mesentery vein wall damage, Alexa Fluor 647-labeled PECAM-1 antibody (Alexa Fluor 647 anti-mouse CD31 antibody, BioLegend, United States) was administered into the femoral vein. To visualize the vessel wall and platelets, 3,3′-dihexyloxacarbocyanine iodide (DiOC6(3), 0.1 mM in 0.05 ml of the mixture of dimethyl sulfoxide (DMSO) and phosphate-buffered saline (PBS) (1:50 volume ratio); Life Technologies, Molecular Probes, United States) was administered via intramuscular injection 5 min before thrombosis induction. Then, a midline laparotomy incision was made, and the mesentery of the ileum was then pulled out of the abdomen and draped over a plastic mound. The mesentery vein was microscopically examined and identified. The mesentery was continuously perfused with prewarmed (37 °C) PBS to prevent drying of the vessels. The mesentery vein wall was injured using a 532-nm argon ion ablation laser (Ablate™, Intelligent Imaging Innovations, Inc., United States). The induction and the progression of thrombosis were recorded for 3 min. One record was divided into 25 time points. At each time point, the area of the thrombus was encircled. The sum of the values of the thrombus area from each time point was referred to as the total thrombus area. PECAM-1 is an antithrombotic molecule present on the platelet surface. The number of activated platelets decreases with an increase in PECAM-1 in the thrombus^21^. To assess the activity of platelets in the thrombus, the area of fluorescence of PECAM-1 was also measured at each time point. Then, the area of PECAM-1 fluorescence at a particular time point was divided by the thrombus area at that time point. The values from one time point were added and referred to as the PECAM-1/thrombus ratio. In each mouse, one thrombus was induced. Representative images of the thrombus and PECAM-1 present on the platelet surface are shown in the Supplementary Fig. 1.

### Platelet activation index assessed using flow cytometry

CytoFLEX Model A00-1-1102 (Beckman Coulter Inc., Unites States) with CytExpert 2.4 (Beckman Coulter Inc., Unites States)^22^. was used in the study. Mouse blood was centrifuged at 129×*g* for 10 min at room temperature. The platelet count in the supernatant was estimated (10 × 10^7^/ml) using a ScilVet ABC Plus + hematological analyzer (HORIBA ABX, France). The platelet suspension was mixed with Alexa Fluor 647-labeled annexin V (final dilution 1:15, Thermo Fisher Scientific, United States), which directly binds to phosphatidylserine (PS, a marker of irreversibly activated, procoagulant platelets), and Dylight 647‐labeled antiplatelet antibodies (CD42b (glycoprotein Ib: GPIb), final concentration 0.066 mg/ml, Emfret Analitics, Unites States). Then, the platelets were supplemented with CaCl_2_ (final concentration 1 mM), activated with adenosine diphosphate (ADP, final concentration 20 µM), and gated based on the binding of anti-GPIb antibodies. Among resting platelets, two distinct platelet populations (P1 and P2) were identified, which differed in the GPIb expression. Platelets with a higher GPIb expression (P2 in the present study) are more prone to be activated^23,24^. After activation with ADP the population of procoagulant platelets with PS and with an increased level of GPIb (P3 population in our study) was formed^23^. Platelet activity was assessed based on the platelet activation index, which was determined by dividing the percentage of total events of P2 before activation by the percentage of total events of P2 after activation (see Supplementary Fig. 2). A high platelet activation index indicates that, during activation, the number of platelets in the P2 population was decreased, and a large amount of platelets were passed to the P3 population as a result of potent activation or to the P1 population as a result of desensitization. A low platelet activation index indicates that, during activation, some platelets were passed from P1 to P2, but platelets from P2 were not activated enough to express PS.

Procoagulant platelets also exposed P-selectin which is a marker of platelet granule secretion^25,26^. In our study we confirmed that PS positive platelets exposed also P-selectin (Supplementary Fig. 3).

### Evaluation of fibrin net density in mouse platelet-poor plasma (PPP)

Fibrin net density in clots was evaluated as described previously^20^ with some modifications. In brief, mouse blood PPP was obtained by centrifuging whole blood at 14,000×*g* for 20 min. Alexa Fluor 488-labeled human fibrinogen (Fibrinogen from Human Plasma, Alexa Fluor™ 488 Conjugate, Thermo Fisher Scientific, United States) was added at a final concentration of 15 µM to the samples of PPP. To induce clot formation, CaCl_2_ was added at a final concentration of 20 mM. The samples were then incubated at 37 °C for 1 h. Relative clot density was determined from the images of the resultant clots. In each image, five 50-µm-long straight lines were placed randomly. The number of fibrin fibers crossing each line was counted. The average of the resultant values was referred to as fibrin net density.

### Euglobulin clot lysis time (ECLT)

ECLT was used to determine the time of clot dissolution. It was measured following the method of Tomczyk et al.^27^ with some modifications^20^. To obtain the euglobulin fraction, 100 µl of PPP was mixed with 190 µl of 0.016% acetic acid. The resulting mixture was incubated for 1 h at 4 °C and then centrifuged (4000 rpm for 10 min at 4 °C). The obtained pellet (euglobulin fraction) was dissolved in 100 µl of Tris buffer, pH 7.4. Of the euglobulin fraction, 100 µl was kept in a well of a microplate, and clot formation was initiated by adding 33 µl of CaCl_2_ solution in Tris buffer, pH 7.4, to the euglobulin fraction (final CaCl_2_ concentration of 10 mM in the well). The changes in optical density in the wells were recorded at a wavelength of 405 nm at 37 °C using the microplate factor ELx808 at 1-min intervals. In the recordings, ECLT was defined as the time from adding CaCl_2_ solution to the well (initial absorbance value) to achieving the minimal absorbance value due to clot lysis.

### Statistical analysis

The data obtained were analyzed using GraphPad Prism 5^28^. The normality of the data distribution was assessed using the Shapiro–Wilk test. Differences between the groups were assessed using one-way ANOVA (for normally distributed data) with the appropriate post hoc test or the Mann–Whitney U test (for non-normally distributed data). For paired observations, the Wilcoxon test was used (non-normally distributed data). The results were expressed as mean ± SEM (for normally distributed data) or as median (with interquartile range, for non-normally distributed data) of the number of determinations (*n*). A *p*-value of < 0.05 was considered significant.

### Approval for animal experiments

Experiments were conducted in accordance with the EU Guidelines on Animal Experiments (European Directive 2010/63/EU). All procedures involving animals and their care were approved by the Local Ethical Committee on Animal Testing in Olsztyn (Approval No.: 81/2021).

## Results

### General characteristics of the animals

The presented changes in the body weight refer to changes between the 14th (14 days after STZ or citrate buffer injection) and 28th day (28 days after STZ or citrate buffer injection) of the experiment in each group. Weight gain was observed in the Contr Norm-female and Contr Norm-male groups. A decrease in body weight was observed in Contr STZ-female and Contr STZ-male groups. Body weight was found to be decreased in the CANA-treated Norm-female group. In the CANA-treated STZ-female group, the decrease in body weight was comparable to that observed in the Contr STZ-female group. Decreased body weight was also observed in the CANA-treated STZ-male group, but as with the STZ-female groups, this decrease was comparable to that in the Contr STZ-male group. DAPA decreased the weight of the STZ-female and STZ-male groups. The decrease was comparable to that in the Contr STZ groups.

Only 38% of the Contr STZ-female group were found to be diabetic 14 days after the STZ injection (14th day of the experiment, first day of drugs or gum arabic administration), whereas 100% was achieved in the Contr STZ-male group. However, 28 days after the STZ injection, both Contr STZ-female and Contr STZ-male groups were found to be diabetic. CANA did not affect the blood glucose level. DAPA increased the blood glucose level in the Norm and STZ-female groups. However, in the DAPA STZ-female group, the increase was comparable to that in the Contr STZ-female group. DAPA did not affect the blood glucose level in male mice.

An increase in the blood level of βHB was observed (at 28th day of experiment) only in the CANA-treated STZ-female group in comparison with Contr STZ-female group.

The general characteristics of mice is presented in Table 1.

**Table 1:** General characteristics of mice. *F* female, *M* male, *Contr* control, *STZ* diabetes, *Norm* normoglycemia. *14 th day* 14th day of the experiment, the day when the CANA and DAPA administration started, 14 days after STZ or citrate buffer injection; *28th day* 28th day of the experiment, the last day of CANA and DAPA administration, 28 days after STZ or citrate buffer injection. ^#^*p* < 0.05, ^###^*p* < 0.001 vs Norm Contr 28th day, *p < 0.05, **p < 0.01, ***p < 0.001 vs 14th day, ^$^*p* < 0.05 vs STZ Contr 28th day; *n* = 10–14.

Day	F Norm Contr		F STZ Contr		F Norm CANA		F STZ CANA		F Norm DAPA		F STZ DAPA	
	14th	28th	14th	28th	14th	28th	14th	28th	14th	28th	14th	28th
Weight (g)	22 (21; 22)	23 (22; 23)*	21 (20; 21)	19 (19; 20)***^,#^	20 (19; 21)	18 (17; 19)***^,###^	21 (20; 21)	18 (17; 19)***	22 (22; 23)	22 (21; 22)	20 (19; 20)	18 (17; 19)***
Blood glucose level (mg/dl)	86 (80; 110)	100 (92; 105)	155 (138; 391)	452 (397; 499)***^,###^	108 (98; 118)	117 (106; 130)	143 (117; 238)	231 (152; 274)	95 (90; 105)	226 (126; 257)***	136 (121; 325)	327 (269; 349)***
βHB (mmol/l)	1.2 (1.03; 1.38)		1.1 (0.8; 1.25)		1.75 (1.3; 2.33)		2.1 (1.85; 2.85)^$^		1.3 (1.05; 1.55)		1.3 (1.15; 1.7)	

Day	M Norm Contr		M STZ Contr		M Norm CANA		M STZ CANA		M Norm DAPA		M STZ DAPA	
	14th	28th	14th	28th	14th	28th	14th	28th	14th	28th	14th	28th
Weight (g)	22 (21; 23)	23 (22; 25)**	21 (20; 22)	18 (17; 19)***^,#^	21.5 (21; 23)	21 (20; 23)	21.5 (20; 24)	16.5 (16; 18)**	22 (21; 23)	22 (21; 23)	21 (21; 21.5)	19 (18; 20)**
Blood glucose level (mg/dl)	116 (104; 125)	110 (103; 119)	380 (318; 460)	482 (406; 499)*	111 (105; 120)	112 (101; 144)	425 (400; 484)	353 (290; 429)	112 (105; 114)	115 (106; 135)	438 (332; 498)	386 (298; 462)
βHB (mmol/l)	1 (0.93; 1.25)		0.8 (0.73; 0.8)		1.7 (1.5; 2.2)		0.6 (0.6; 0.7)		1.2 (0.9; 1.3)		0.8 (0.7; 1.08)	

### Laser-induced thrombosis in the mesenteric vein and the assessment of platelet activity based on the PECAM-1/thrombus ratio

**Figure 1:**
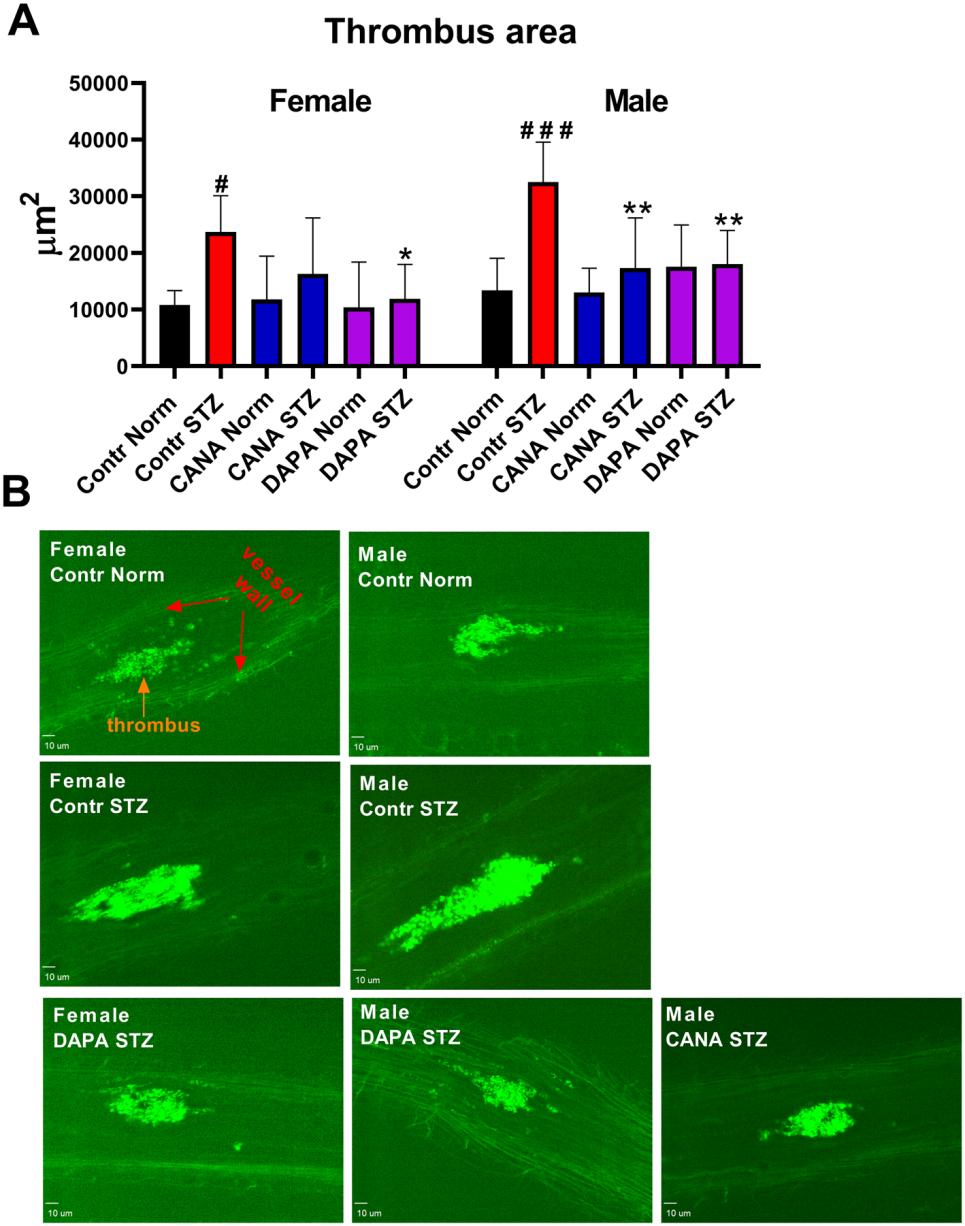
The effects of CANA and DAPA on laser-induced thrombosis. (**A**) The effects of DAPA and CANA on the thrombus area. (**B**) Representative confocal microscopy images of thrombi. Thrombus and the vessel wall have been indicated. Bar = 10 µm. Pictures were captured with SlideBook 6.0^19^. ^#^*p* < 0.05, ^###^*p* < 0.001 vs Contr Norm, *p < 0.05, **p < 0.01 vs Contr STZ; *n* = 7–14.

**Figure 2:**
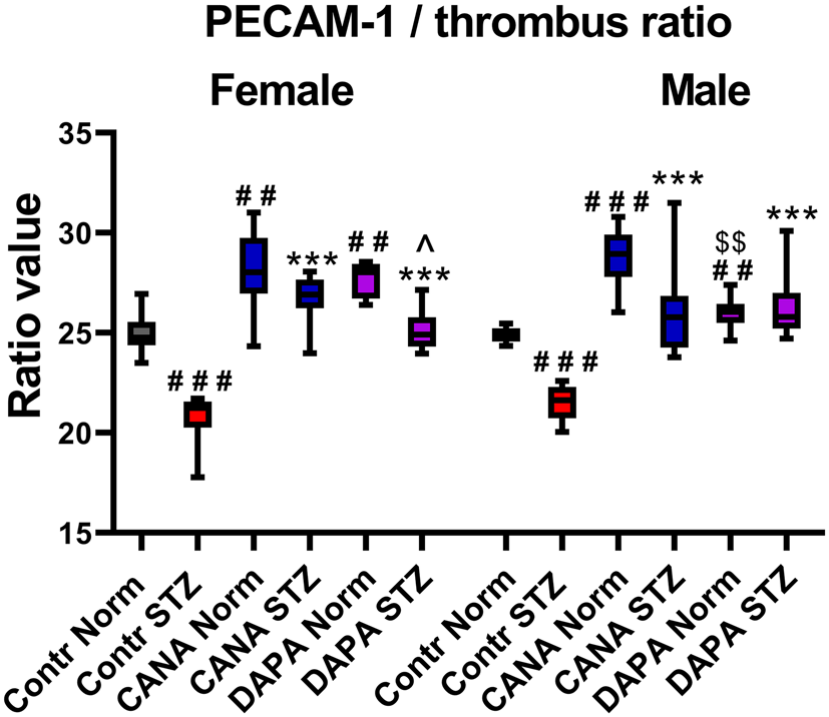
The effects of CANA and DAPA on the PECAM-1/thrombus ratio. ^##^*p* < 0.01, ^###^*p* < 0.001 vs Contr Norm, ***p < 0.001 vs Contr STZ, ^*p* < 0.05 vs Female CANA STZ, ^$$^*p* < 0.01 vs Male CANA Norm; *n* = 7–14.

To determine the effects of CANA and DAPA on the thrombotic process and the platelet activity in the thrombus, laser-induced thrombosis was carried out. Hyperglycemia increased the thrombus area in the Contr STZ-female and Contr STZ-male groups. CANA decreased the thrombus area only in the STZ-male group. DAPA decreased the thrombus area in the STZ-male and STZ-female groups (Fig. 1). Images of vessels before ablation are presented in Supplementary Fig. 4. In the Contr STZ-female and Contr STZ-male groups, a decreased PECAM-1/thrombus ratio was observed. CANA and DAPA increased the PECAM-1/thrombus ratio in all tested groups (Fig. 2).

### Platelet activation index assessed using flow cytometry

The platelet populations was gated on the expression of the GPIb which is a specific platelet surface marker. GPIb fluorescence was plotted as a function of forward scatter (FSC, which roughly corresponds with the cells size^29^). Before activation we identified two platelet populations with the same FSC and different GPIb fluorescence but the GPIb (platelet staining) intensity is similar in all groups (see Supplementary Fig. 5).

**Figure 3:**
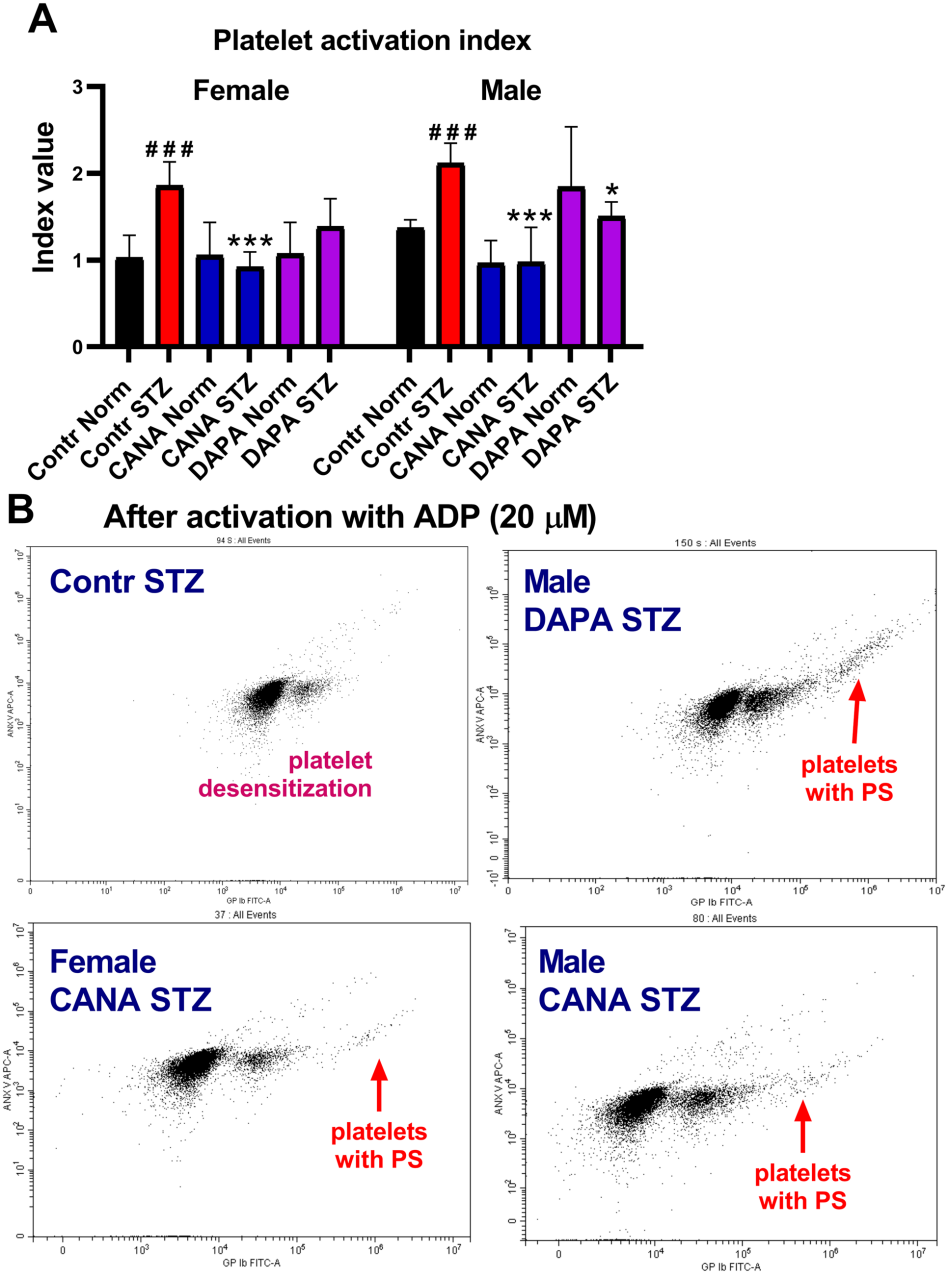
Assessment of the platelet activation index using flow cytometry. (**A**) The effects of CANA and DAPA on the platelet activation index. (**B**) Representative images from flow cytometry, which show platelet populations after ADP treatment. Red arrows indicate the appearance of the P3 population (platelets with PS). Dot plots were captured with CytExpert 2.4^22^. ^###^*p* < 0.001 vs Contr Norm, *p < 0.05, ***p < 0.001 vs Contr STZ; *n* = 7–10.

To evaluate the effects of DAPA and CANA on platelet activity, the platelet activation index was evaluated after platelet activation with ADP. In the Contr STZ-female and Contr STZ-male groups, the platelet activation index was increased. DAPA decreased the platelet activation index only in the STZ-male group, whereas CANA decreased it in the STZ-female and STZ-male groups (Fig. 3). In the Contr STZ-female and Contr STZ-male groups, platelets were desensitized, and after ADP treatment, no P3 population appeared. The platelet response to ADP was restored by CANA in the STZ-female and STZ-male groups and DAPA in the STZ-male group, which was expressed by the appearance of the P3 population (Fig. 3B).

### Evaluation of fibrin net density in mouse PPP

**Figure 4:**
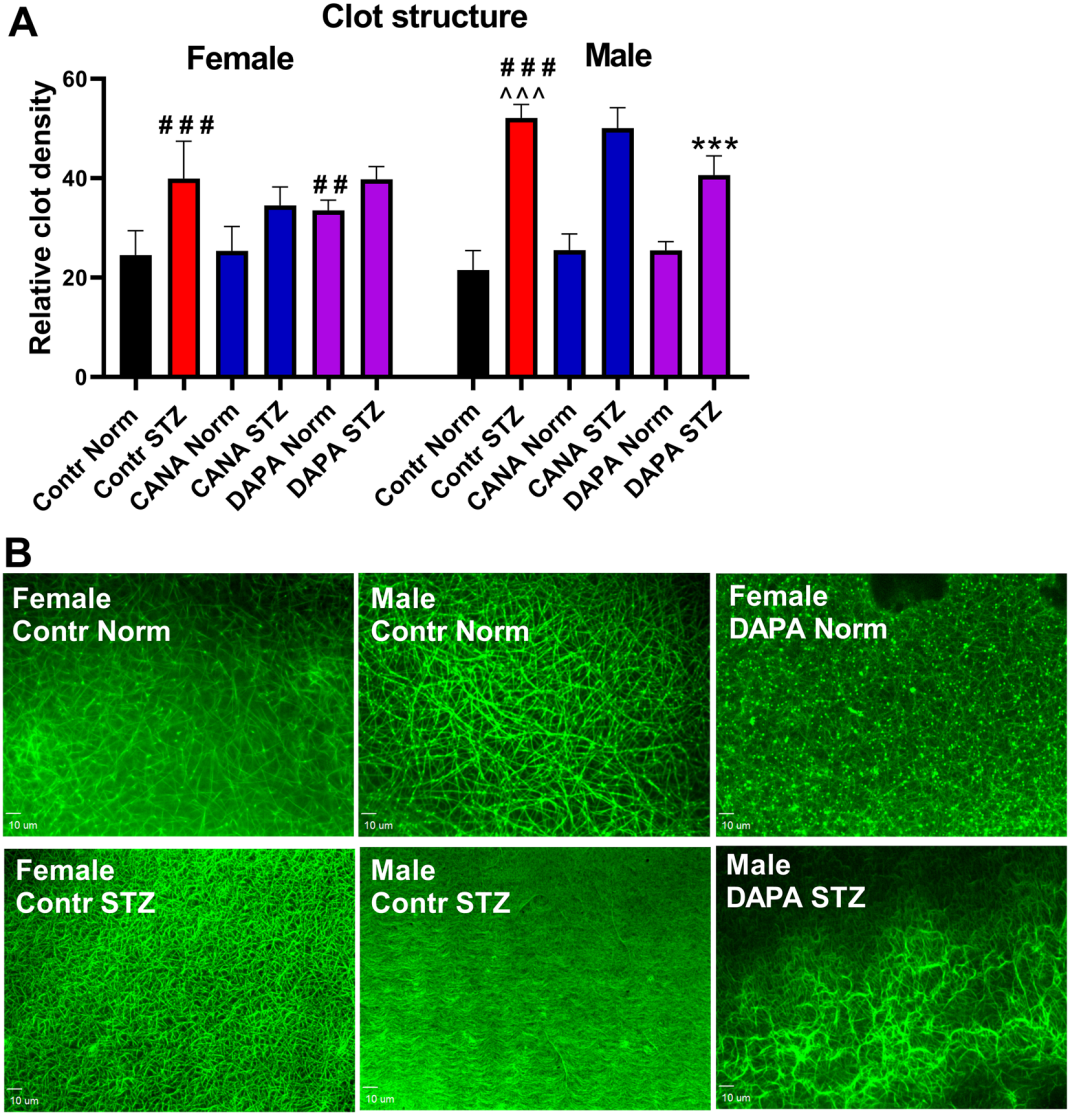
The effects of CANA and DAPA on fibrin net density. (**A**) The effects of CANA and DAPA on fibrin net density in clots formed after recalcination of PPP. (**B**) Representative confocal microscopy images of fibrin net. Bar = 10 µm. Pictures were captured with SlideBook 6.0^19^. ^##^*p* < 0.01, ^###^*p* < 0.001 vs Contr Norm, ***p < 0.001 vs Contr STZ, ^^^*p* < 0.001 vs Female Contr STZ; *n* = 7–9.

To investigate the effects of CANA and DAPA on the coagulation system, fibrin formation was induced in PPP. The Contr STZ-male group showed a higher fibrin net density than the Contr STZ-female group, most likely due to prolonged hyperglycemia. CANA did not affect fibrin formation. DAPA increased fibrin net density in the Norm-female group and decreased it in the STZ-male group (Fig. 4).

### ECLT

**Figure 5:**
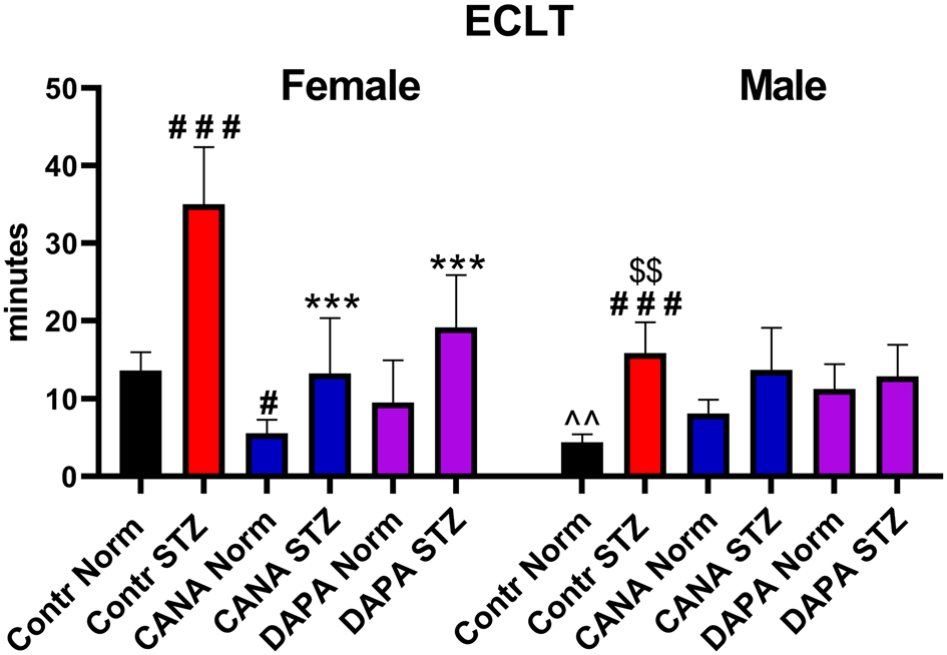
The effects of CANA and DAPA on euglobulin clot lysis time. ^#^*p* < 0.05, ^###^*p* < 0.001 vs Contr Norm, ***p < 0.001 vs Contr STZ, ^^*p* < 0.01 vs Female Contr Norm, ^$$^*p* < 0.01 vs Female Contr STZ; *n* = 7–11.

To evaluate the effects of CANA and DAPA on fibrinolysis, ECLT was calculated. In the Contr male groups, ECLT was shorter than in the Contr female groups, both Norm and STZ. Hyperglycemia prolonged ECLT in the Contr STZ-female and Contr STZ-male groups. CANA shortened ECLT in the Norm and STZ-female groups. DAPA shortened ECLT only in the STZ-female group. CANA and DAPA did not change ECLT in male mice (Fig. 5).

## Discussion

CANA and DAPA exerted sex-specific effects on hemostasis. They both exerted a beneficial antithrombotic effect, which was more pronounced in male mice. The antithrombotic mechanism could be attributable to platelet inhibition as indicated by an increased PECAM-1/thrombus ratio and decreased platelet activity after ADP. The effects of CANA and DAPA on plasma coagulation and fibrinolysis are not clear since an increased activity of the coagulation system and activation of fibrinolysis were observed only in female mice.

In addition, the effects of CANA and DAPA were investigated in two different states of hemostasis: nonactivated in normoglycemia and activated by hyperglycemia^20^. STZ-induced diabetes models were used to achieve such high hyperglycemia and to make the effects of SGLT2i independent of their hypoglycemic activity. However, the lack of an increase in the blood glucose level between 14 and 28th day of the experiment in CANA STZ-female, CANA STZ-male, and DAPA STZ-male gropus as in the case of Contr STZ groups may indicate hypoglycemic effects of tested drugs. Despite this, the animals from abovementioned groups remain hyperglycemic throughout the experiment. Therefore, it is unlikely that the observed changes were related to the hypoglycemic effect of the drugs, because glucose levels did not normalize. CANA and DAPA effects were more pronounced in hyperglycemia and included attenuation of the thrombotic process, platelet inhibition, reduction in coagulation activity, and increase in fibrinolysis activity, with no hypoglycemic effect. However, the beneficial effect of SGLT2i in normoglycemia was limited to a slight inhibition of platelet activity (female and male mice) and activation of fibrinolysis (only in female mice). Furthermore, unfavorable effects were observed in normoglycemic female mice treated with DAPA, as indicated by the increased fibrin net density. Since glycemia affects fibrin formation^30^, this effect could be partially due to the increased glucose level, probably as a result of increased glucagon production^31^. More pronounced effects on activated hemostasis may explain the fact that DAPA reduced the risk of major adverse cardiovascular events in patients with prior MI and thus in patients with activated hemostasis, whereas it had no effect in patients without prior MI^3^. Hemostasis activation in men after MI was manifested as increased VIII and X coagulation factors, enhanced ADP-induced aggregation, and attenuated fibrinolysis^32^.

Platelets play a key role in the process of thrombus formation during stroke or MI^6^. Many studies show that diabetes contributes to increase of platelet activity. In the group of patients with type 1 diabetes (T1D) with good and poor glycemic control the enhanced expression of P-selectin and increased number of platelet-monocyte aggregates were observed^33^. Conditions of vessels affect platelet activity in T1D. It has been shown that an increase in the expression of P-selectin on thrombin-activated platelets was observed only in patients with microangiopathies^34^. Furthermore, increased expression of P-selectin on ADP-activated platelets was observed in patients with T1D with concomitant nephropathy, while expression of P-selectin on platelets derived from patients without nephropathy was compared with P-selectin expression in healthy volunteers^35^. Platelets in type 2 diabetes (T2D) are characterized with enhanced secretion of P-selectin and exposure of glycoprotein IIb/IIIa which determine the ability of platelet to aggregation^36^. Similar to T1D, in T2D, the condition of the vessels affects the activity of platelets. Impairment of endothelial function in T2D leads to insufficient release of NO and prostacyclin, which are potent platelets inhibitors^37^. This shows how important is not only hypoglycemic effect of antidiabetic drugs but also their pleiotropic effects.

In the laser-induced thrombosis model, platelets aggregated at the site of the vascular injury. The area of the thrombus formed in this model was dependent on the platelet activity, platelet–subendothelial matrix interaction, and platelet–endothelium interaction. Exposed areas of the subendothelial matrix in this model were small; thus, potent and irreversible activation did not occur. Platelets that formed the thrombus in this model did not expose PS, thus enabling the investigation of platelet activity in the aggregation state^38^. Platelet activity in this state was assessed using the PECAM-1/thrombus ratio. PECAM-1 is a transmembrane glycoprotein of the immunoglobulin family and is present, among others, on the platelet surface^39^. It is an antithrombotic molecule, and it has been reported that PECAM-1 knockout mice exhibit a prothrombotic phenotype^40^. As shown in our previous study, the PECAM-1/thrombus ratio can more precisely describe the platelet activation status in mice (intravital) and humans (in vitro) than the commonly used parameters such as thrombus area^21^. Therefore, an increase in PECAM-1 in the thrombus (increased PECAM-1/thrombus ratio) results in less activated platelets^21^. Platelet activity was assessed also based on PS expression after ADP treatment as the platelet activation index. During activation, platelets change their shape, and PS, which is located in the inner leaflet of the plasma membrane of resting platelets, is irreversibly translocated to the outer leaflet. PS catalyzes the activation of coagulation factors; therefore, platelets with exposed PS are called procoagulant^41^. Assessment of the surface PS expression is a common method for determining platelet procoagulant activity^42^. Conversion of non-procoagulant platelet to procoagulant platelet is a complex process that involves molecular pathways similar to that occurring in apoptotic cells like cytoskeletal rearrangement or PS exposure. It has been shown that processes that lead to agonist-induced PS exposure are distinct from those regulating platelet apoptosis. Apoptotic platelets can be also procoagulant but this conversion is independent of agonist action and granule secretion^26^. In our study platelet procoagulant activity was measured before and after ADP activation (Supplementary Fig. 2). In our experimental conditions the population with PS is only visible after platelet stimulation with ADP which is a platelet agonist. As it was shown in the Supplementary Fig. 3 platelets with PS exposed also P-selectin which is a marker of platelet granule secretion^25^. This indicates that the P3 population in our experiment shows procoagulant platelets.

In the present study, platelets from the STZ Contr groups were desensitized due to high endogenous ADP concentrations and did not respond to ADP stimulation; thus, no platelets with PS (in the present study P3 population) were observed after activation. This finding is consistent with the clinical observation in which platelets of patients with hyperglycemia were preactivated and stimulation with ADP did not result in platelet activation or the appearance of activation markers^43^. CANA (in female and male mice) and DAPA (in male mice) reversed this process and partially restored the platelet response to ADP regardless of glycemic control.

In STZ-male mice, the antiplatelet effect of CANA and DAPA (expressed as a decreased platelet activation index and an increased PECAM-1/thrombus ratio) translated into the antithrombotic effect, but this trend was not observed in female mice. Potent platelet inhibition (expressed as an increased PECAM-1/thrombus ratio and a decreased platelet activation index) did not translate into the antithrombotic effect in STZ-female mice after CANA treatment. This indicates that despite the antiplatelet effect, the interactions between platelets and the subendothelial matrix or endothelium were not attenuated in female mice as it was in male mice. Furthermore, CANA is associated with an increased risk of ST-elevation myocardial infarction (STEMI) and a lower risk of non-STEMI (NSTEMI)^44^. In most cases of STEMI, the thrombus is primarily composed of fibrin, platelets, and erythrocytes^45^, whereas in NSTEMI, there is usually a large proportion of platelets in the thrombus^46^. In the abovementioned study in a group of patients with NSTEMI, men constituted 72.2% and patients who experienced NSTEMI and had preexisting cardiovascular diseases constituted 80.3%. In the present study, since a decrease in thrombus formation and platelet activity was observed in male mice with activated hemostasis, it can be assumed that the reduction in the risk of NSTEMI could be partially explained by the antiplatelet activity of CANA. However, due to the only beneficial effect of CANA demonstrated in this study, the increased risk of STEMI may be attributable to other reasons than impairment of hemostasis. It is important to note that DAPA displayed a weaker antiplatelet effect in STZ-female mice than CANA, which was expressed as a lower PECAM-1/thrombus ratio and no effect on the platelet activation index. Despite this, a reduction in thrombus formation was observed STZ-female mice after DAPA treatment. This indicates that the antithrombotic effect of DAPA in this group was not dependent only on platelet inhibition. It can be concluded that the mechanisms of antithrombotic activity of CANA and DAPA are different, multifaceted, and dependent on sex. This could stem from the different biological activity and also from the influence on sex hormones^47^.

This might be confirmed by a few studies that evaluated the mechanism of SGLT2i on platelets. There are reports of the involvement of the platelet Na^+^/H^+^ exchanger (NHE) in the antiplatelet effect of SGLT2i. It has been demonstrated that DAPA in vitro inhibited the activation of NHE on human platelets^48^. However, the effect of CANA on platelet NHE has not been evaluated, but it has been demonstrated that CANA inhibits NHE in cardiomyocytes^49^. Na^+^ influx through platelet NHE is essential to platelet activation^50^. An enhanced platelet NHE activity in diabetic patients contributes to increased platelet activation, which shows inhibition of NHE as a potential therapeutic antiplatelet strategy. An increased expression and activity of NHE and, therefore, more target sites may contribute to the fact that the antithrombotic effects of CANA and DAPA were observed in hyperglycemia. The activity toward NHE could explain the sex-dependent effects of CANA and DAPA since the expression and activity of NHE are sex dependent and change through the estrus cycle^51,52^. Different activities of CANA and DAPA toward platelets could stem from their different effects on heme oxygenase 1 (HO-1). It has been reported that CANA, but not DAPA, increases HO-1 activity in smooth muscle cells^53^. HO-1 breaks down heme into biliverdin and carbon monoxide (CO). HO-1 deficiency is observed in vascular diseases (e.g., atherosclerosis)^54^, and its decreased activity contributes to endothelial dysfunctions in hyperglycemic rats^55^. Furthermore, CO, which is a product of HO-1 activity, inhibits platelet activation^56^. CANA may exert its antiplatelet effect through the increasing activity of HO-1 and the subsequent CO release.

CANA decreased fibrin net density only in STZ-male mice. In addition, DAPA showed opposite effects on the female and male coagulation systems since it increased fibrin net density in Norm-female mice but decreased it in STZ-male mice. As mentioned earlier, the enhancement of fibrin formation after treatment with DAPA might be due to the increased blood glucose level. The blood glucose level is positively associated with fibrinogen concentration in patients with and without diabetes^57^, and increased glycemia increases fibrinogen glycation and increases the overlap among fibrin fibers in clot^58^. However, other mechanisms could be involved. It has been demonstrated that DAPA exerted beneficial effect on the coagulation system under impaired hemostasis, and attenuated platelet-independent production of thrombin, which is a part of the coagulation system, and its activity is crucial in the process of fibrin formation^9^. This study has been conducted on male *Ldlr*^*−*^*/*^*−*^ mice with hypercholesterolemia, but female mice have not been included. The opposite effect of DAPA on the coagulation system demonstrated in the present study shows that sex differences should be taken into account when assessing the activity of new drugs. Hepatic nuclear factor 4 alpha (HNF4α) is a nuclear transcriptional factor expressed in the liver, pancreas, kidneys, and intestines. DAPA upregulates HNF4α expression in vitro^59^. Since HNF4α directly regulates the transcription of factor XII and factor XIII of coagulation^60^, the upregulation of HNF4α by DAPA may contribute to the increase in fibrin net density in Norm-female mice. HNF4α affects gene expression in a sex-dependent manner^61^, and it could be the reason why the increase in fibrin net density was observed only in female mice treated with DAPA. Furthermore, DAPA normalizes the levels of luteinizing hormone and partially restores ovulation in obese female mice^47^, which may indicate the sex-hormone-related effect of DAPA.

Sex-dependent effects of SGLT2i were observed in relation to the production of βHB. The use of SGLT2i is associated with an increased risk of euglycemic ketoacidosis in normoglycemic patients and type 2 diabetes patients. Euglycemic ketoacidosis is a complex acid–base disturbance with a normal or slightly increased blood glucose level^62^. DAPA was used as an adjunct therapy in type 1 diabetes patients, but it has been withdrawn as it increased the risk of euglycemic ketoacidosis. The decrease in the blood glucose level after treatment with DAPA in type 1 diabetes was often associated with a reduction in the insulin dose, which leads to an increase in the glucagon level. This causes the metabolism shift toward the oxidation of fatty acids and promotes the production of ketone bodies such as βHB, whose increased concentration contributes to acidosis^62^. In the present study, an experimental type 1 diabetes model was used. Hyperglycemia in the STZ-induced model of diabetes results from β cell dysfunctions since STZ destroys β cells located in the large islets in the pancreatic core, whereas the remaining β cells show insufficient and impaired insulin production^63^. Furthermore, an increased number of α cells and an increased blood glucagon level have been reported^64^. Diabetic ketoacidosis is a major acid–base disturbance in type 1 diabetes and, unlike euglycemic ketoacidosis, is associated with uncontrolled hyperglycemia^65^. Because of the lack of insulin treatment in the present study, the animals were under persistent hyperglycemia, but the level of the βHB remained unchanged. Literature data also indicate that hyperglycemia in STZ-induced diabetic mice is not always associated with the increased level of βHB^66,67^. In the present study, only STZ-female mice treated with CANA exhibited an increased level of βHB, which is consistent with clinical data^62,68^. Fibrinolysis differed between Contr male and female mice, both normoglycemic and hyperglycemic, which indicates that fibrinolysis is sex dependent regardless of the glycemic condition^69–71^. The improvement in fibrinolysis after both CANA and DAPA administration was observed only in female mice. However, whether the observed differences were due to the sex-specific effect of the drug or the influence of sex on the fibrinolysis process remains unclear. The fact that women are more likely to develop ketoacidosis^62^ and CANA is the most prone to induce ketosis among SGLT2i^68^ could have influenced the effect of CANA on fibrinolysis. βHB has been reported to activate eNOS and NO release in rat arteries^72^. Since NO affects fibrinolysis^73^, the reduction in ECLT in female mice after CANA treatment may be partially dependent on the increased production of βHB and subsequent NO release^74^. Furthermore, the pharmacokinetics of CANA could have contributed to its different effect on fibrinolysis as CANA exposure in women and female mice is higher than in men and male mice^75,76^. However, the reason for the activated fibrinolysis in STZ-female mice after DAPA treatment and the possible mechanism of this effect could not be found.

Due to the protective effect of estrogens, the susceptibility of β cells to STZ is lower in female than in male mice^77^. Therefore, in the present study, the progression of diabetes differed between male and female mice. Only 38% of the Contr STZ-female group had a blood glucose level higher than 200 mg/dl 14 days after STZ injection, whereas 100% was achieved in the Contr STZ-male group. However, 28 days after STZ injection, blood glucose levels in the Contr STZ-female and Contr STZ-male groups were comparable, which shows that both male and female mice developed diabetes. Therefore, differences in fibrin net density and ECLT between the Contr STZ-female and Contr STZ-male groups could stem from the disparate severity of diabetes. Distinct conditions of glucose homeostasis may have contributed to the different characteristics observed in drug effects between STZ-male and STZ-female mice. Despite this limitation, the findings of this study are relevant because this is the first study to show that DAPA and CANA affect activated hemostasis in uncontrolled hyperglycemia.

Based on the findings of this study, it can be concluded that DAPA and CANA exert sex-specific and multidirectional effects on particularly impaired hemostasis beyond glycemic control. Although SGLT2i have been used with antithrombotic drugs, side effects such as bleeding have not been reported so far, whereas a reduction in cardiovascular risk has been observed^44,78^. This could indicate that the use of SGLT2i with an antithrombotic drug does not lead to an increased risk of bleeding but normalizes hemostasis. Less pronounced favorable effects of CANA and DAPA in normoglycemia may be beneficial in patients with high cardiovascular risk without previous thrombotic events. However, clinical studies are necessary to confirm this. This study also showed the importance of sex-specific studies to identify the most effective therapy and avoid sex-specific side effects.

## Supplementary Information

Supplementary Information.

## Data Availability

All datasets generated and analyzed in the current study are available from the corresponding author upon reasonable request.

## Abbreviations

ADP: Adenosine diphosphate
βHB: β Hydroxy butyrate
CANA: Canagliflozin
CANVAS: Canagliflozin Cardiovascular Assessment Study
CO: Carbon monoxide
Contr: Control
DAPA: Dapagliflozin
DECLARE-TMI 58: Dapagliflozin Effect on Cardiovascular Events
DMSO: Dimethyl sulfoxide
ECLT: Euglobulin clot lysis time
EDGE: The Effect of Dapagliflozin on Platelet Function Testing Profiles in Diabetic Patients
eNOS: Endothelial nitric oxide synthase
FSC: Forward scatter
GPIb: Glycoprotein Ib
HNF4α: Hepatic nuclear factor 4 alpha
HO-1: Heme oxygenase 1
MI: Myocardial infarction
NHE: Na^+^/H^+^ exchanger
NO: Nitric oxide
Norm: Normoglycemic
NSTEMI: Non-STEMI
PBS: Phosphate-buffered saline
PS: Phosphatidylserine
PECAM-1: Platelet endothelial cell adhesion molecule 1
PPP: Platelet-poor plasma
SGLT2i: Sodium-glucose cotransporter 2 inhibitors
STEMI: ST-elevation myocardial infarction
STZ: Streptozotocin
T1D: Type 1 diabetes
T2D: Type 2 diabetes
