# Supplementary material for: Sex-dependent effects of canagliflozin and dapagliflozin on hemostasis in normoglycemic and hyperglycemic mice
Source: Sci Rep. 2023 Jan 17;13:932. doi: 10.1038/s41598-023-28225-8 (PMC9845220; doi:10.1038/s41598-023-28225-8)
Supplement: Supplementary file 1 — Supplementary Information. [file 41598_2023_28225_MOESM1_ESM.pdf]

Sex-dependent effects of canagliflozin and dapagliflozin on hemostasis in normoglycemic and hyperglycemic mice

Natalia Marcińczyk [1*], Tomasz Misztal [2], Ewa Chabielska [1], Anna Gromotowicz-Popławska [1]

1 Department of Biopharmacy and Radiopharmacy, Medical University of Bialystok, 15-222 Białystok, Poland

2 Department of Physical Chemistry, Medical University of Bialystok, 15-089 Bialystok, Poland

*Correspondence: Natalia Marcińczyk, natalia.marcinczyk@umb.edu.pl

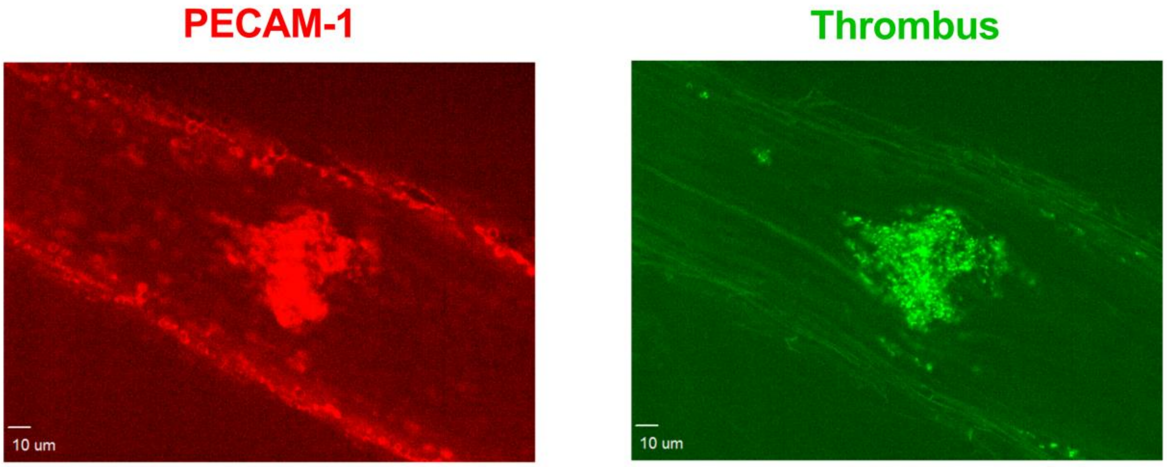

Supplementary Figure 1. Representative pictures of the thrombus (green) and PECAM-1 (red) present on the platelet surface.

Before activation

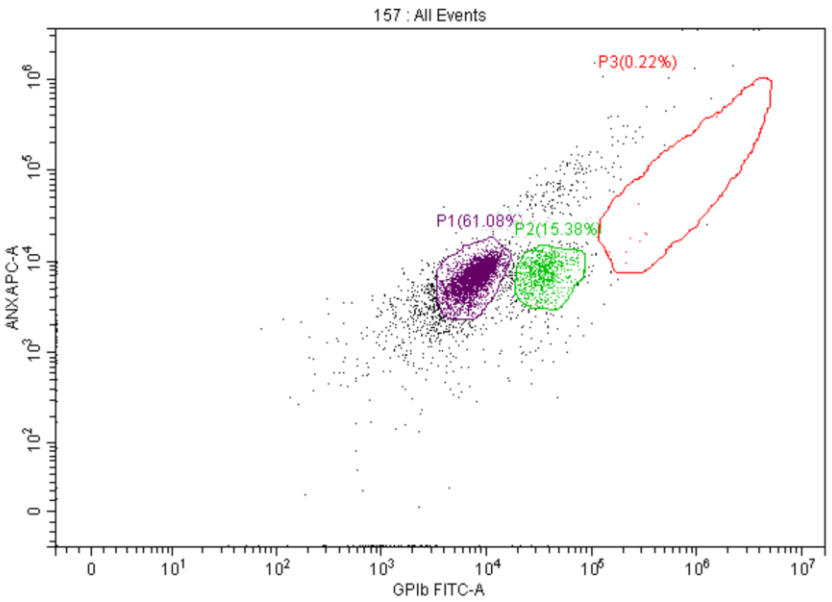

After activation with ADP (20 μM)

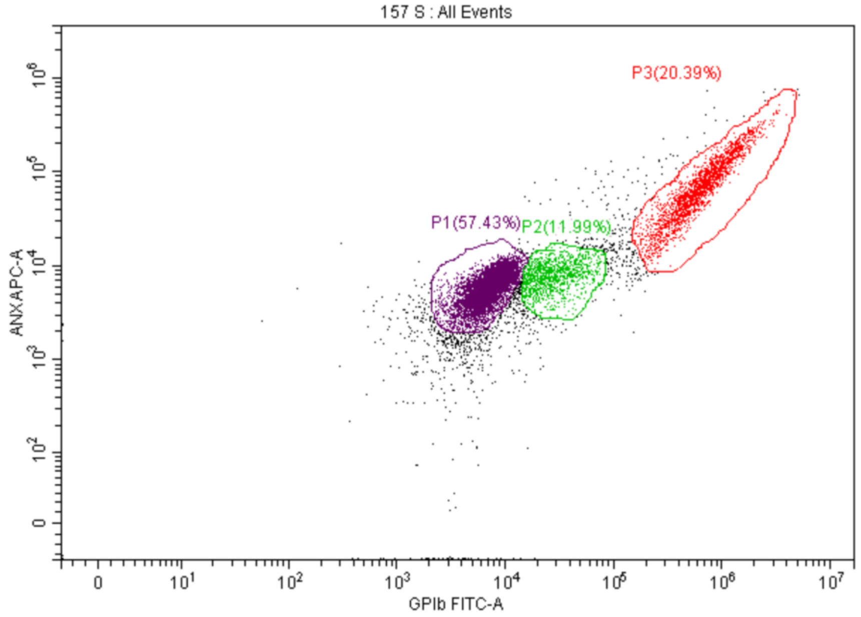

Supplementary Figure 2. Representative platelet populations in male normoglycemic mice. Each population is encircled. The top panel shows platelet populations before activation, whereas the bottom panel shows platelet populations after activation.

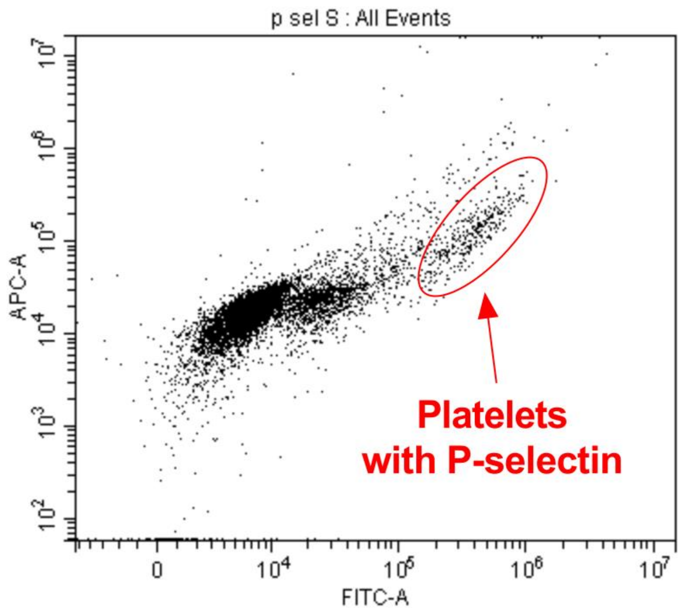

Supplementary Figure 3. Image of dot plot from flow cytometer. In the dot plot the platelet population with exposed P-selectin is encircled.

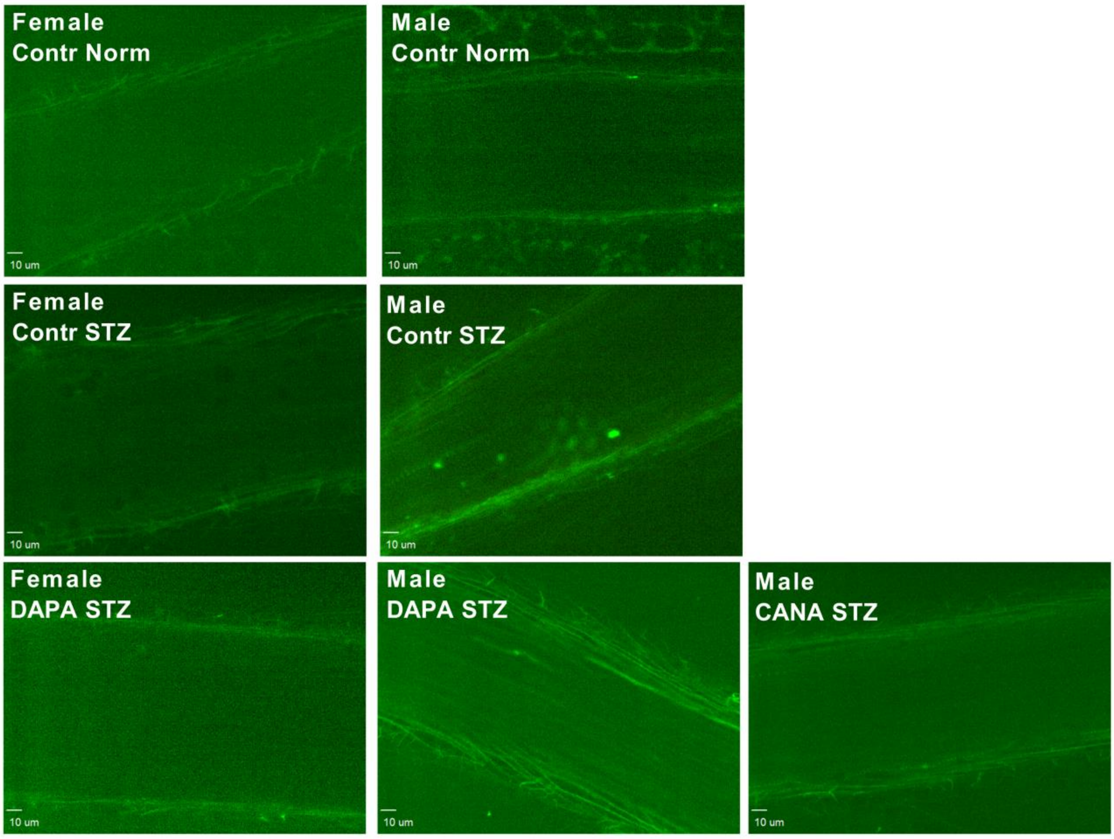

Supplementary Figure 4. Images of vessels before ablation. Figure shows vessels in which thrombi presented in Figure 1 were induced.

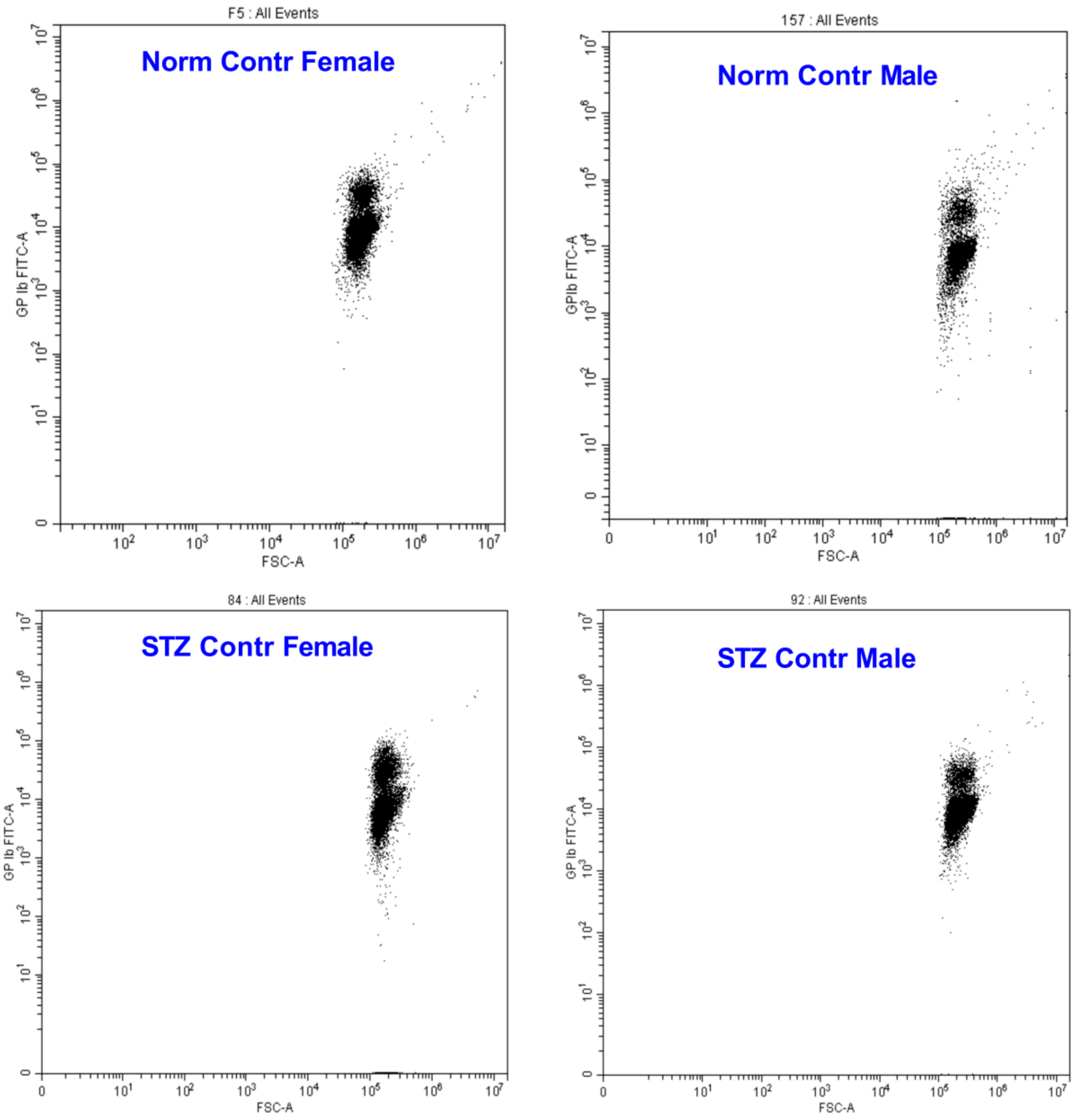

Supplementary Figure 5. GPIb fluorescence was plotted as a function of FSC. Each dot plot shows two platelet populations before activation with ADP.
